# Mechanically-sensitive miRNAs bias human mesenchymal stem cell fate via mTOR signalling

**DOI:** 10.1038/s41467-017-02486-0

**Published:** 2018-01-17

**Authors:** Jessica E. Frith, Gina D. Kusuma, James Carthew, Fanyi Li, Nicole Cloonan, Guillermo A. Gomez, Justin J. Cooper-White

**Affiliations:** 1 Materials Science and Engineering, Monash University, Clayton, VIC 3800 Australia; 2 Australian Institute for Bioengineering and Nanotechnology, University of Queensland, St Lucia, 3072 Australia; 3 School of Biological Sciences, The University of Auckland, Auckland Central, Auckland, 1010 New Zealand; 4 Centre for Cancer Biology, SA Pathology and the University of South Australia, Frome Road, Adelaide, 5000 SA Australia; 5 Institute for Molecular Bioscience, Cell Biology, University of Queensland, St Lucia, QLD 3072 Australia; 6 School of Chemical Engineering, University of Queensland, St Lucia, QLD 3072 Australia; 7 Biomedical Manufacturing Manufacturing Flagship, CSIRO, Clayton, VIC 3169 Australia

## Abstract

Mechanotransduction is a strong driver of mesenchymal stem cell (MSC) fate. In vitro, variations in matrix mechanics invoke changes in MSC proliferation, migration and differentiation. However, when incorporating MSCs within injectable, inherently soft hydrogels, this dominance over MSC response substantially limits our ability to couple the ease of application of hydrogels with efficiently directed MSC differentiation, especially in the case of bone generation. Here, we identify differential miRNA expression in response to varying hydrogel stiffness and RhoA activity. We show that modulation of miR-100-5p and miR-143-3p can be used to bias MSC fate and provide mechanistic insight by demonstrating convergence on mTOR signalling. By modulating these mechanosensitive miRNAs, we can enhance osteogenesis in a soft 3D hydrogel. The outcomes of this study provide new understanding of the mechanisms regulating MSC mechanotransduction and differentiation, but also a novel strategy with which to drive MSC fate and significantly impact MSC-based tissue-engineering applications.

## Abstract (web summary)

Mesenchymal stem cell (MSC) fate can be mechanically regulated by substrate stiffness but this is difficult to control in a 3D hydrogel. Here the authors identify miRNAs that change expression in response to substrate stiffness and RhoA signalling and show that they can bias MSC fate in a 3D soft hydrogel.

## Introduction

Mesenchymal stem cells (MSCs) are attractive candidates for tissue engineering, as a result of their availability, immunosuppressive properties and ability to differentiate into bone, cartilage, fat and other stromal cell types^1,2^. When combined with tailored biomaterial systems, such as bio-synthetic hydrogels, MSC-based tissue-engineering strategies have tremendous potential to revolutionise the manner by which we repair and regenerate damaged tissues including bone, cartilage, tendon and intervertebral disc. However, in reality, without methods for efficient differentiation of MSCs within these biomaterials, it is difficult to ensure formation of the desired tissue type. This currently presents a major challenge for the field, and our ability to direct MSC differentiation must be improved in order to successfully generate functional new tissue. Improving our knowledge of MSC-biomaterial interactions and how such interactions determine fate specification, as well as developing new strategies that utilise this knowledge to successfully drive cell fate within hydrogels, will help realise their potential for tissue generation.

It is well established that MSCs are highly sensitive to physical stimuli from their surrounding microenvironment, including matrix viscoelastic properties^3,4^, topography^5^, extracellular matrix (ECM) specificity^6^ and mode of ligand presentation^7^. In particular, substrate stiffness (elastic modulus) has received significant attention and has proven to be an important cue in directing MSC proliferation and differentiation, with soft substrates favouring differentiation into adipocytes or neurons while stiff substrates promote osteoblast differentiation^4,8,9^. Such cues highlight the vital role that biomaterial cues may play when optimising the differentiation of MSCs in composites for tissue engineering.

The common link between the various physical stimuli that impact upon MSC fate is their ability to alter MSC mechanotransductive signalling and subsequent morphology via the RhoA, Rac and Cdc42 GTPases. These GTPases regulate cytoskeletal and focal adhesion composition and function, associated changes in cell spreading and morphology, and ultimately impact cell fate^10^. The role of such mechanotransductive signalling in MSC fate was first established by McBeath et al.^11^. who demonstrated that highly spread MSCs were osteogenic, rounded MSCs were adipogenic and that the osteo-adipogenic fate switch was mediated by differences in RhoA activity. Subsequently, Rac1 activity has been reported to direct a similar switch between well-spread smooth muscle cells and rounded chondrogenic MSCs in the presence of TGFβ3^12^. In the presence of BMP2, inhibition of Rac1 activity has also been shown to promote MSC osteogenesis^13^. Rac1 was also shown to be critical in regulating the impacts of variations in substrate viscoelasticity on MSC fate, including changes in lineage specification, cytoskeletal stress and mitosis^14^.

Manipulation of mechanical signalling offers the opportunity to direct MSC differentiation towards a particular lineage and enhance the efficiency of conversion to a specified mature cell type for tissue-engineering applications. However, traditional methods of signalling modulation, such as growth factors and small molecule inhibitors may prove difficult to translate effectively into clinical treatments, due to their lack of specificity for the target cell, stability and cost. For these reasons, modulation of cell behaviour via small oligonucleotides, including microRNAs (miRNAs), is an attractive proposition. miRNAs, a class of small regulatory RNAs (~22 bp), offer a relatively simple, inexpensive and scalable way to guide cellular activities. They regulate gene expression by either changing mRNA stability or inhibiting protein translation^15^, and in doing so, modulate the activity of several target genes and converge upon signalling/regulatory networks^16^, helping to generate an extremely robust biological output.

miRNAs play important roles in directing MSC fate, including proliferation, osteogenesis, adipogenesis and chondrogenesis^17–20^. Interestingly, the pro-osteogenic effect of *miR-138* was mediated by its effects upon focal adhesion kinase (FAK), suggestive of a link between miRNA signalling, MSC mechanotransduction and MSC fate^21^. There are also many further indications of miRNAs acting at all levels of the mechano-regulatory hierarchy, from ECM proteins to integrins, focal adhesion and cytoskeletal components, as well as RhoA, Rac, Cdc42 and the guanine nucleotide exchange factors and GTPase-activating proteins (GAPs) that regulate their activity^22,23^. However, the potential of using miRNAs to modulate MSC mechanotransductive pathways and subsequent fate has not been addressed to date.

In this study, we investigate the role of miRNA signalling in response to variations in substrate stiffness and evaluate how modulation of mechanosensitive miRNAs can be used to drive MSC fate in injectable hydrogels. We identify and validate a panel of miRNAs that are differentially expressed both in response to changing substrate stiffness and RhoA activity, and further demonstrate that modulation of these candidate miRNAs can alter MSC osteo-adipogenic differentiation bias. We provide an insight into the mechanism of action by showing that *miR-100-5p* and *miR-143-3p* converge on mTOR signalling. Finally, we prove that modulation of these mechanosensitive miRNAs can significantly enhance osteogenesis in a 3D soft hydrogel, confirming the utility of this novel approach. Together the outcomes of this study provide new insight into the regulatory role of miRNAs in mechanotransduction and suggest a novel application of miRNAs in harnessing mechanical signalling to successfully drive MSC differentiation towards osteogenesis in soft hydrogels. This strategy can be applied to a multitude of other tissue targets, and thus is seen to have significant potential to impact MSC-based tissue-engineering applications.

## Results

### MSC properties in response to substrate stiffness and RhoA

**Fig. 1:**
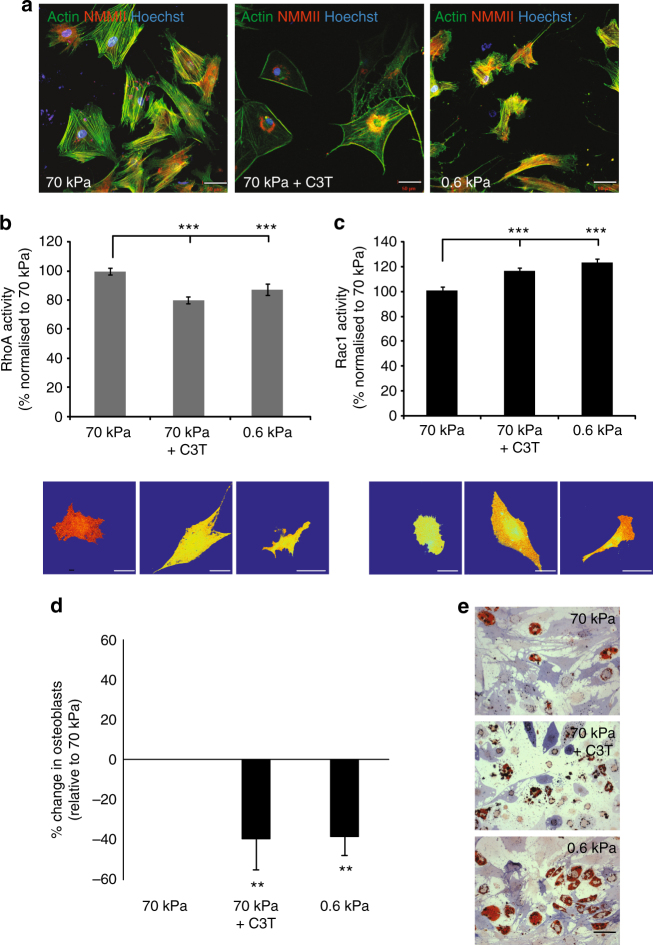
Characterisation of hMSC properties in response to substrate stiffness and RhoA inhibition. **a** Morphology of hMSCs showing actin (green), nuclei (blue) and non-muscle myosin II (red). Scale bar, 50 μm. **b** RhoA and **c** Rac1 activity, as determined by FRET biosensor. Data is shown as mean ± SEM for hMSCs from three independent donors (*n* > 20/donor) with heatmaps of a representative cell. Scale bar, 50 μm. **d** Differentiation switch between osteogenesis and adipogenesis. Graph shows mean percentage change in osteoblasts ± SD for *N* = 4 MSC donors. **e** Representative images of alkaline phosphatase (blue) and Oil Red O (red) staining of hMSCs under the three different conditions. Scale bar, 100 μm. Samples were analysed by one-way ANOVA with Tukey post hoc testing. Statistically different samples are denoted by *p < 0.05, **p < 0.01 and ***p < 0.001

Using polyacrylamide (PAM) gels functionalised with collagen-1, we first characterised the properties of hMSCs in response to varying substrate stiffness and RhoA inhibition. hMSCs were seeded onto stiff and soft gels with a Young’s modulus of 70 and 0.6 kPa, respectively (Supplementary Fig. 1). Given our hypothesis that high RhoA activity on stiff substrates and low RhoA activity on soft substrates is a critical mediator of the hMSC response to substrate stiffness, in a third variant, hMSCs on stiff substrates were treated with C3T, a potent and specific RhoA inhibitor. There were evident changes to hMSC morphology in response to these alterations, with hMSCs on stiff substrates being well-spread with highly aligned and thick actin fibres, while those on the soft substrates were much smaller and highly elongated, with narrow and more randomly oriented actin fibres. hMSCs treated with C3T showed a different morphology and were highly spread, with a polygonal shape and actin fibres localised predominantly to the periphery of the cell body (Fig. 1a). Image analysis confirmed these changes in cell spread area, aspect ratio and circularity (Supplementary Fig. 2).

Determination of RhoA activity using FRET biosensors confirmed that RhoA activity was significantly decreased both by the C3T inhibitor and in hMSCs on soft substrates, as compared to MSCs on stiff substrates (Fig. 1b). Using a Rac1 biosensor^14^, we showed that conversely Rac1 activity was elevated in these hMSC populations (Fig. 1c). To determine what impact these changes had on hMSC fate, cells under our three model conditions were treated with a 1:1 mixture of osteogenic and adipogenic supplements. Given all of the necessary soluble factors for both osteogenic and adipogenic differentiation, hMSCs on the stiff substrates predominantly became osteogenic, while hMSCs on the soft substrates, or treated with C3T, were biased towards adipogenesis. This was evident by the significant reduction in the percentage of osteoblasts in hMSC populations treated with C3T, or on 0.6 kPa substrates as compared to MSCs on 70 kPa substrates (Fig. 1d, e).

### miRNA expression in response to substrate stiffness and RhoA

**Fig. 2:**
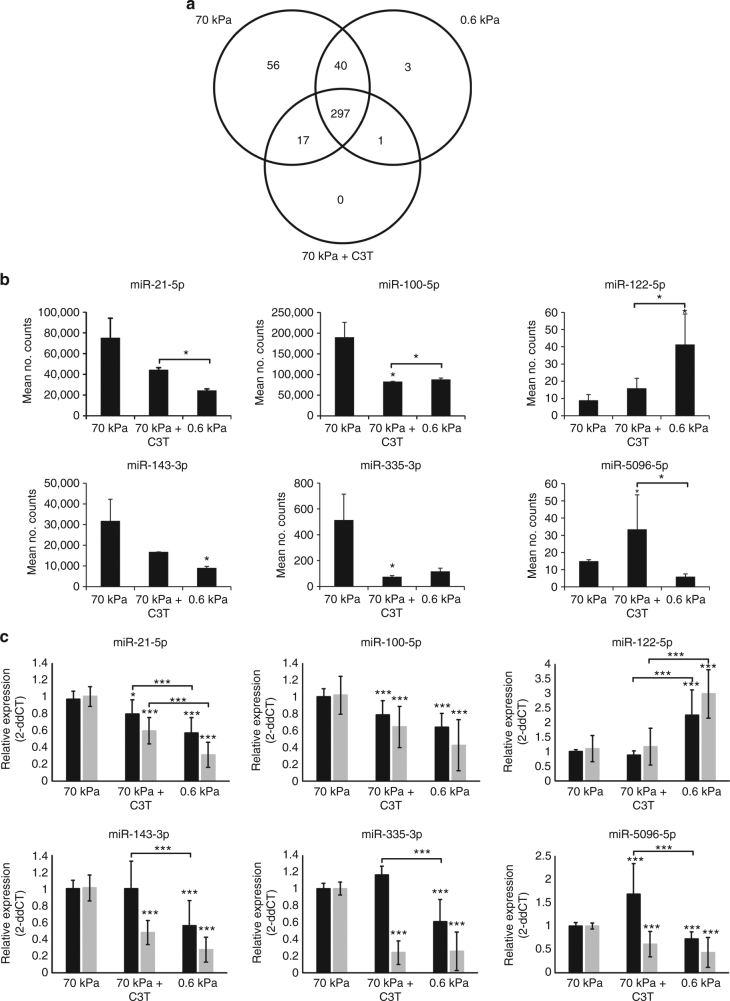
Changes to miRNA expression in response to substrate stiffness and RhoA activity. **a** Venn diagram showing overlap of detected miRNAs between samples **b** Mean counts from miRNA sequencing ± SEM. **c** qPCR validation of miRNA expression at 24 h (black) and 7 days (grey). Data is shown relative to expression in 70 kPa samples. All data is represented as mean ± SD for four independent hMSC donors. Separate experiments were sampled for sequencing and qPCR. Samples were analysed by one-way ANOVA with Tukey post hoc testing. Statistically different samples are denoted by *p < 0.05, **p < 0.01 and ***p < 0.001

To determine whether there are differences in miRNA expression in response to substrate stiffness or RhoA activity, miRNA sequencing was performed on samples from three independent hMSC donors using cells cultured under the three conditions (70 kPa, 70 kPa + C3T and 0.6 kPa) for 24 h. Within each condition, approximately 400 miRNAs were expressed and differences were observed both in the presence or absence of specific miRNAs (Fig. 2a) as well as the expression level of many miRNAs (Fig. 2b, Table 1 and Supplementary Tables 1–9). Clustering of the samples indicated that the C3T-treated cells shared similarities with both cells on 70 and 0.6 kPa substrates, with two samples clustering with cells from 70 kPa substrates and one clustering within the 0.6 kPa substrate set (Supplementary Fig. 3).

**Table 1:** Summary of miRNA sequencing data

Comparison (A:B)	Upregulated (in B vs. A)	Downregulated (in B vs. A)	Unchanged
70 kPa vs. 70 kPa + C3T	14	4	506
70 kPa vs. 0.6 kPa	2	0	522
70 kPa + C3T vs. 0.6 kPa	11	27	486
70 kPa vs. [70 kPa + C3T and 0.6 kPa]	5	1	518
[70 kPa and 70 kPa + C3T] vs. 0.6 kPa	17	6	501

For each differentially expressed miRNA, candidate target mRNAs were predicted from Targetscan and analysed using Ingenuity Pathway Analysis. Supplementary Data 1–3 list all the 'canonical pathways' showing significant enrichment in the lists of these target genes. Overlap was observed between the lists, including for many pathways associated with mechanotransduction (Actin Cytoskeleton Signaling, HIPPO signalling, FAK signalling, Integrin signalling) and particularly RhoA and GTPase signalling (Regulation of Actin-based Motility by Rho, RhoA Signaling, RhoGDI Signaling, Rac signalling, Cdc42 signalling), as well as some of the major developmental signalling pathways (BMP, FGF, Wnts) and other pathways known to be involved in regulation of MSC fate (Adipogenesis pathway, PPAR signalling, Role of Osteoblasts, Osteoclasts and Chondrocytes in Rheumatoid Arthritis, mTOR Signaling).

We selected a panel of miRNA candidates for further investigation, focussing on candidates that showed the greatest difference in expression between the conditions, as well as those being expressed at moderate to high levels of counts in the sequencing data (Fig. 2b). Validation of differentially expressed miRNAs was performed by qPCR using samples independent to those used for sequencing and showed the same trends as the sequencing data (Fig. 2b, c). A further comparison with expression levels after 7 days culture showed that these trends were largely maintained and often more stark at this later timepoint (Fig. 2c).

### Modulation of miRNA signalling can influence MSC fate

Given the large difference in hMSC lineage specification induced by substrate stiffness and RhoA activity, we next determined what impact the modulation of these differentially expressed miRNAs might have on the differentiation bias of hMSCs. hMSCs were transfected with miRNA mimics or inhibitors, cultured on both soft and stiff substrates in mixed induction medium and the change in bias between osteogenesis and adipogenesis determined.

**Fig. 3:**
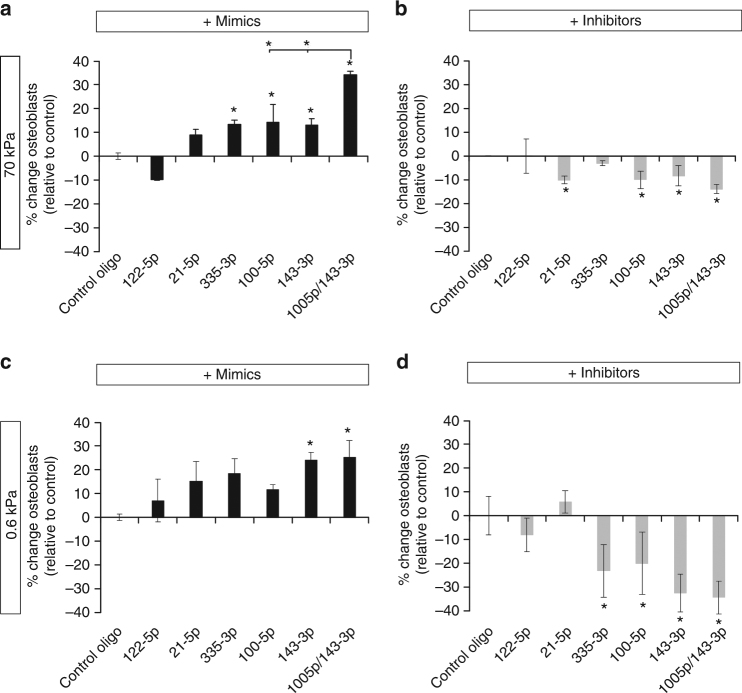
Modulation of miRNA activity can modulate the hMSC differentiation bias in response to substrate stiffness. Differentiation bias of hMSCs treated with **a** miRNA mimics or **b** miRNA inhibitors and cultured on 70 kPa gels and **c** treated with miRNA mimics or **d** miRNA inhibitors and cultured on 0.6 kPa gels. Data is shown as mean ± SD of the change in proportion of osteoblasts (see Supplementary Fig. 4 for additional donors). Samples were analysed by one-way ANOVA with Tukey post hoc testing. Statistically different samples are denoted by (*)

Across both 70 and 0.6 kPa substrates, no consistent effect on hMSC fate bias was observed for *miR-122-5p*, *miR-21-5p* or *miR-335-3p*. However, mimics of *miR-100-5p* and *miR-143-3p*, both of which were expressed at greater levels in stiff substrates, caused a significant increase in the proportion of osteoblasts when compared to cells treated with a control oligonucleotide (Fig. 3a, c; Supplementary Fig. 4). On the 70kPa substrate, combining mimics of both *miR-100-5p* and *miR-143-3p* had a larger effect than modulating either miRNA individually. Inhibition of these miRNAs had the opposite effect on hMSC differentiation bias to the mimics, with *miR-100-5p*, *miR-143-3p* and both *miR-100-5p*, and *miR-143-3p* together causing a significant decrease in the proportion of osteoblasts in the culture (Fig. 3b, d).

### Mechanosensitive miRNAs modulate MSC fate via mTOR pathway

**Fig. 4:**
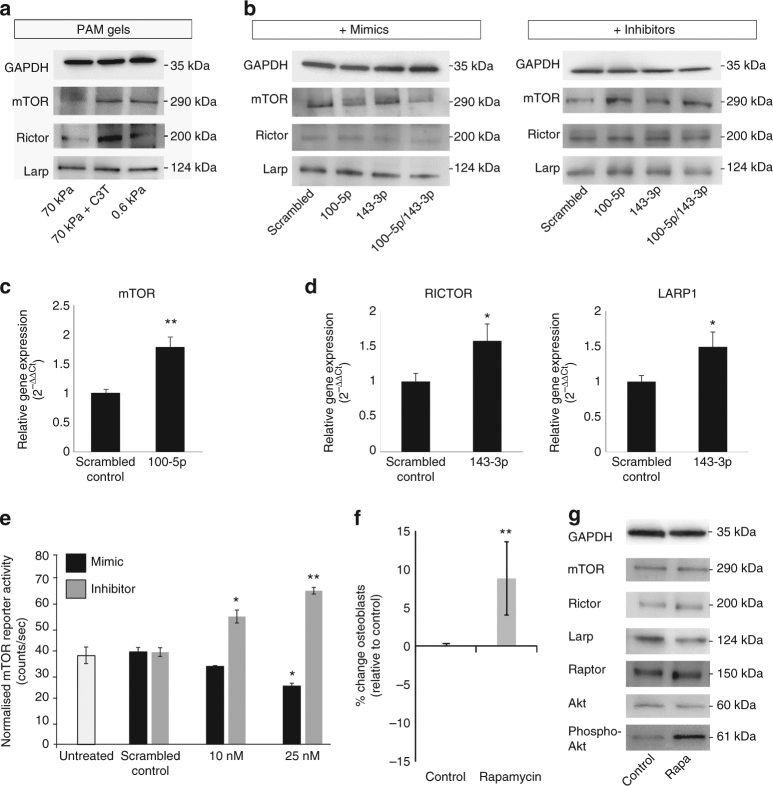
*miR-100-5p* and *miR-143-3p* converge on mTOR signalling. **a** Western blots of miRNA targets in response to substrate stiffness. **b** Western blots of miRNA targets in hMSCs treated with miRNA mimics and inhibitors. **c** qPCR determination of the relative expression of miRNA target genes in hMSCs treated with miRNA inhibitors for *miR-100-5p* and **d** *miR-143-3p*. Samples were analysed by one-way ANOVA with Games-Howell post hoc testing. **e** 3′UTR reporter assay for FRAP1 (mTOR) in hMSCs transfected with mimics (black) and inhibitors (grey) of *miR-100-5p*. **f** Differentiation switch assay of MSCs treated with 50 nM rapamycin. Data is shown as mean ± SD of the change in proportion of osteoblasts. **g** Western blots of mTOR pathway components in hMSCs treated with 50 nM rapamycin. All graphs show mean ± SD for three independent hMSC donors relative to expression in control samples. Samples were analysed by one-way ANOVA with Tukey post hoc testing. Statistically different samples are denoted by *p < 0.05, **p < 0.01 and ***p < 0.001

A closer analysis of the predicted targets of *miR-100-5p* and *miR-143-3p*, both of which showed the most significant impact upon the differentiation of hMSCs, revealed a potential convergence upon mTOR signalling, with *miR-100-5p* targeting *mTOR* itself, while *RICTOR* and *LARP*, both key components of the mTOR network, were predicted targets of *miR-143-3p*. Western blotting showed upregulation of mTOR and Rictor with C3T treatment and on 0.6 kPa substrates- the conditions in which we observed decreased expression of *miR-100-5p* and *miR-143-3p* (Fig. 4a, Supplementary Fig. 5A). These changes were mirrored by changes in the expression of mRNA for these genes, suggesting that regulation of transcript levels may be the mechanism via which these changes in protein expression are achieved (Supplementary Fig. 5B and C). There was no apparent difference in Larp1 protein levels, although an increase in *LARP1* was also detected at the mRNA level.

Confirming the regulation of these mTOR components within our system, transfection of hMSCs with a mimic of *miR-100-5p* decreased mTOR levels, while co-transfection of *miR-100-5p* and *miR-143-3p* mimics reduced Rictor and Larp1 protein levels. Conversely, mTOR and Rictor expression was elevated in MSCs treated with inhibitors of *miR-100-5p*, *miR-143-3p* or both and Larp1 levels were increased in the presence of *miR-143-3p* mimic (Fig. 4b; Supplementary Fig. 6A). qPCR verified that these changes were likely caused by upregulation of transcript levels for mTOR. Likewise, treatment with *miR-143-3p* inhibitor increased RICTOR and Larp1 transcript levels (Fig. 4c, d).

To provide functional validation of the interaction of *miR-100-5p* with mTOR, we used a dual luciferase/alkaline phosphatase (ALP) reporter system to probe the interaction of *miR-100-5p* with the 3′UTR of *FRAP1* (mTOR). Scrambled control oligonucleotides did not alter reporter activity from untreated levels, but *miR-100-5p* mimics and inhibitors significantly decreased and increased reporter activity respectively, thus confirming a direct interaction between *miR-100-5p* and mTOR regulation in our cells (Fig. 4e).

We next treated hMSCs with rapamycin, an inhibitor of mTOR, to determine whether this would have similar effects to downregulation of mTOR components mediated by *miR-100-5p* and *miR-143-3p*. In a differentiation bias assay, rapamycin caused a similar effect to *miR-100-5p*/*143-3p* mimics and enhanced the bias of differentiation towards osteogenesis (Fig. 4f). Western blotting confirmed the inhibition of mTORC1 with 50 nM rapamycin, by an increase in phosphorylation of Akt at serine 473. Under this inhibition by rapamycin, we observed no change to mTOR, or Raptor protein levels, an increase in Rictor and a decrease in Larp1 expression (Fig. 4g, Supplementary Fig. 6B).

**Fig. 5:**
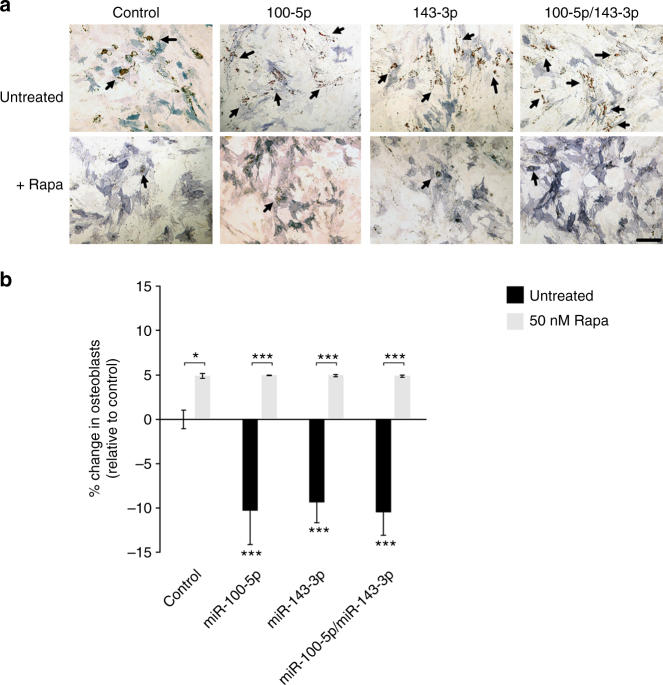
Rapamycin over-rides the effect of miRNA inhibitors. **a** Representative images of alkaline phosphatase (blue) and Oil Red O (red) staining of hMSCs transfected with miRNA inhibitors with and without 50 nM rapamycin. Scale bar, 100 μm. **b** Differentiation bias of hMSCs treated with miRNA inhibitors with (grey bars) and without (black bars) 50 nM rapamycin. Data is shown as mean ± SD of the change in proportion of osteoblasts (see Supplementary Fig. 7 for additional donors). Samples were analysed by one-way ANOVA with Tukey post hoc testing. Statistically different samples are denoted by *p < 0.05, **p < 0.01 and ***p < 0.001

To further confirm that mTOR signalling is modulated by *miR-100-5p* and *miR-143-3p*, MSCs were transfected with inhibitors of *miR-100-5p* and *miR-143-3p* and treated with rapamycin. Consistent with our previous results, inhibition of both *miR-100-5p* and *miR-143-3p* enhanced the bias towards adipogenesis resulting in a decrease in the proportion of osteoblasts in the culture (Fig. 5; Supplementary Fig. 7). The addition of 50 nM rapamycin caused a significant change towards osteogenesis. No significant differences were observed between any conditions treated with Rapamycin indicating that inhibition of mTOR signalling is sufficient to overcome differences in differentiation bias caused by inhibition of *miR-100-5p* and *143-3p*. This supports our premise that *miR-100-5p* and *miR-143-3p* exert their effects upon MSC fate via mTOR signalling.

### Modulation of miRNAs to drive MSC fate in 3D hydrogels

Finally, we investigated whether modulation of miRNAs could be used to drive the fate of hMSCs in soft 3D hydrogel constructs (*G*′ = 2.4 kPa)^24^. hMSCs were transfected with miRNA modulators, encapsulated in photo-crosslinkable gelatin-PEG hydrogels^24^ and cultured in the presence of osteogenic supplements for 14 days.

**Fig. 6:**
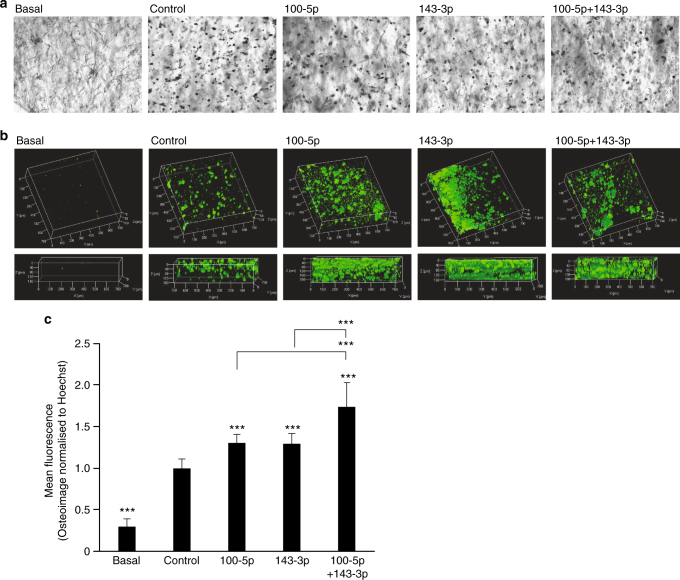
miRNA modulation can enhance MSC differentiation in 3D hydrogels. **a** Phase contrast images of encapsulated hMSCs (black and white) on D14. Scale bar, 20 μm. **b** 3D projections of hydroxyapatite staining (green) after 14 days encapsulation in 3D hydrogels. **c** Quantitation of hydroxyapatite levels. Data is shown as mean Osteoimage intensity relative to Hoechst intensity ± SD for *N* = 9 gels across 3 hMSC donors. Samples were analysed by one-way ANOVA with Tukey post hoc testing. Statistically different samples are denoted by *p < 0.05, **p < 0.01 and ***p < 0.001

After 14 days under osteogenic induction in the hydrogels the hMSCs had extended some processes and many of the cells condensed, becoming dark and granular (Fig. 6a). The gels became more opaque throughout the culture period and in several of the samples, white deposits were visible by eye upon fixation. Osteoimage staining for hydroxyapatite deposition showed mineral accumulation in all gels. Compared to scrambled controls, mimics of *miR-100-5p* and *143-3p* significantly increased the amount of mineral deposition (Fig. 6b, c). This was particularly evident for the samples containing cells transfected with both *miR-100-5p* and *143-3p* mimics which had significantly more mineral deposition than in cultures with either miRNA individually. Almost all of the gel volume was filled with mineral, even at this relatively early culture timepoint (Fig. 6c).

## Discussion

The impact of substrate cues upon MSC fate has been well described, but here we provide the first demonstration that miRNA signalling plays a critical role in regulating the response of hMSCs to their physical microenvironment and that, further to this, modulation of mechanosensitive miRNAs can be used to direct hMSC fate.

Using MSCs cultured on patterned substrates to restrict cell spreading, McBeath et al. previously showed that levels of RhoA activity are determined by changes to MSC shape and that this can bias MSCs towards osteogenesis or adipogenesis^11^. We demonstrated that similar changes to cell spreading, induced by substrate stiffness, can similarly influence RhoA activity and alter the bias towards osteogenic or adipogenic differentiation. Consistent with studies suggesting that RhoA and Rac1 activity act in counterbalance, we observed an increase in Rac1 activity when RhoA activity was decreased^25^. Interestingly, although the differentiation bias of the hMSCs was instructed by both substrate stiffness and RhoA inhibition, the cytoskeletal architecture of MSCs on soft substrates and those treated with C3T was not identical. It is, however, interesting to note that both actin architectures would lead to low levels of tension across the cell, when compared to the cytoskeleton of MSCs on 70 kPa substrates, which further links back to the findings of McBeath et. al. regarding the link between increased cytoskeletal tension, RhoA activity and osteogenic fate^11^. The fundamental reasons for these differences will form an interesting avenue for future studies. However, due to the similarly reduced RhoA activity and subsequent increase in bias towards an adipogenic fate in MSCs treated with C3T, or cultured on soft substrates, we used this model to determine the influence of substrate stiffness and RhoA activity on miRNA signalling in MSC fate.

By deliberately selecting strict criteria regarding the inclusion of miRNAs for analysis, we successfully identified a small number (<100) of miRNAs that showed differential expression between our conditions. Importantly, this strategy allowed us to identify miRNAs with significantly different expression levels, but restricted our analyses to those candidates with expression levels likely to be high enough to exert a significant influence on gene regulation. These findings proved robust, as demonstrated by the close correlation between the miRNAseq data and independent qPCR verification. Although *miR-138*, which regulates osteogenesis in hMSCs has been shown to target *FAK*^21^ and differential expression of miRNAs was reported as part of a study using rat MSC cultures on micro-grooved surfaces^26^, we believe this is the first study to systematically identify changes to hMSC miRNA expression in response to substrate mechanical properties.

Globally, the integration of the miRNAs with mechanotransduction and cell fate regulation was well corroborated by the pathway analysis of the predicted target genes that showed enrichment across pathways involved in cytoskeletal organisation, GTPase signalling and bone or fat formation and maintenance. No systematic differences could be observed in overall pathway contributions when RhoA activity was decreased as a result of either C3T-treatment or soft substrates, with comparisons between all three culture conditions generally acting upon cytoskeletal and Rho/Rac/Cdc42-associated pathways. Despite this, many individual miRNAs were differentially expressed between C3T-treated and soft substrates, perhaps indicative of different routes leading to regulation of these common pathways.

Of the specific miRNA candidates identified here, some have been previously linked to a response to mechanical or matrix cues. This includes *miR-21*, which has been shown to mediate substrate mechanical memory of MSCs^27^, *miR-21*, *miR-100* and *miR-5096* which changed in response to mechanical stretch in human periodontal ligament stem cells^28^ and *miR-494-3p*, which was sensitive to compressive force in MC3T3-E1 cells^29^. *miR-494-3p* was further shown to target *ROCK1*, while *miR-122* has been demonstrated to modulate RhoA and influence motility of hepatocellular carcinoma cells^30^. Many of the miRNAs we identified have also been linked to processes that relate to bone or adipose formation and maintenance^31–33^. This supports our findings that miRNAs in general, and particularly the specific candidates we identified, can regulate both mechanotransductive signalling and MSC differentiation processes.

Of the miRNAs we tested, *miR-100-5p* and *143-3p* had the most influence on hMSC differentiation and were observed to promote osteogenesis. Both *miR-100-5p* and *miR-143-3p* showed a significant decrease in response to C3T-treatment and soft substrates, suggesting that downregulation of their expression is related to low RhoA activity and substrate stiffness. Our findings indicate that both *miR-100-5p* and *miR-143-3p* enhance osteogenesis. Previous reports have suggested that *miR-100* inhibits osteogenesis, which is in contrast to our findings^34,35^. However, these studies used BMP2 as an inductive factor, and demonstrated the inhibitory effect to be mediated by changes to BMPR2^34,35^. In our study, BMP2 was not used as an osteo-inductive factor and so it is possible that this is the reason for the discrepancy^36^. *miR-100* was also upregulated in Lamin A/C knockout MSCs showing additional changes in gene expression that are otherwise suggestive of a switch away from osteogenesis^37^. This study directly links mechanotransduction to the nucleus while we observe changes to cytoplasmic signalling pathways and may highlight the intricacies of force transmission between the cytoplasm and nucleus that are yet to be determined. *miR-143* has no known link to MSC differentiation. However, levels have been shown to increase in differentiating adipocytes and *miR-143* inhibition retards this differentiation^38^. These data are consistent with a role regulating adipogenic differentiation in MSCs, as observed in our study.

Notably, our analysis of putative targets of the miRNAs showed that both *miR-100-5p* and *miR-143-3p* converge upon components of the mTOR signalling network (*mTOR, RICTOR, LARP*), which is known for its critical role in regulating cell fate. Interestingly, mTORC1 regulates *Runx2* and *PPARγ*^39^ and mTORC2 links into regulation of RhoA and cytoskeletal remodelling^40,41^, corroborating the concept that *miR-100-5p* and *143-3p* could regulate both mechanotransductive signalling and hMSC differentiation via mTOR activity. miR-100 has been consistently linked to mTOR in the literature, where it has been shown to regulate proliferation and migration via its effects on mTOR, particularly in cancer cells^42,43^. *miR-143* has also been linked to regulation of mTOR, through inhibition of mTOR and Akt phosphorylation^44^.

We further showed that hMSCs treated with rapamycin, which primarily inhibits mTORC1, showed an increase in osteogenesis, as well as elevated Akt-ser473 phosphorylation. This correlates with a study by Chen et al. indicating that low concentrations of rapamycin induce Akt phosphorylation by inhibiting mTORC1 pathway^45^. This is consistent with studies showing that rapamycin promotes osteogenesis^46^ and that MSCs from mTORC1 (Raptor) knockout mice show an increased osteogenic bias^47^. However, the exact role of rapamycin in osteogenesis can be context-dependent, with studies showing inhibition or promotion of osteogenesis depending upon specific cell type and concentration of rapamycin used^39,48–51^.

The ability of rapamycin to over-ride the effects of *miR-100-5p* and *miR-143-3p* inhibitors provided a clear demonstration that the effects of these factors upon MSC fate is mediated by mTOR signalling. We further showed that *miR-100-5p* targets mTOR itself and so would affect signalling via mTORC1 and mTORC2, while *miR-143-3p* could exert differentiation effects on both mTORC1 and mTORC2 via Larp1 and Rictor, respectively. Although the relative contributions of these miRNAs to overall mTORC1 and mTORC2 bias remains a topic for future investigation, it is clear that *miR-100-5p* and *143-3p* can both directly modulate key mTOR components and cause analogous changes to MSC differentiation, as observed using the small molecule mTOR inhibitor, rapamycin.

**Fig. 7:**
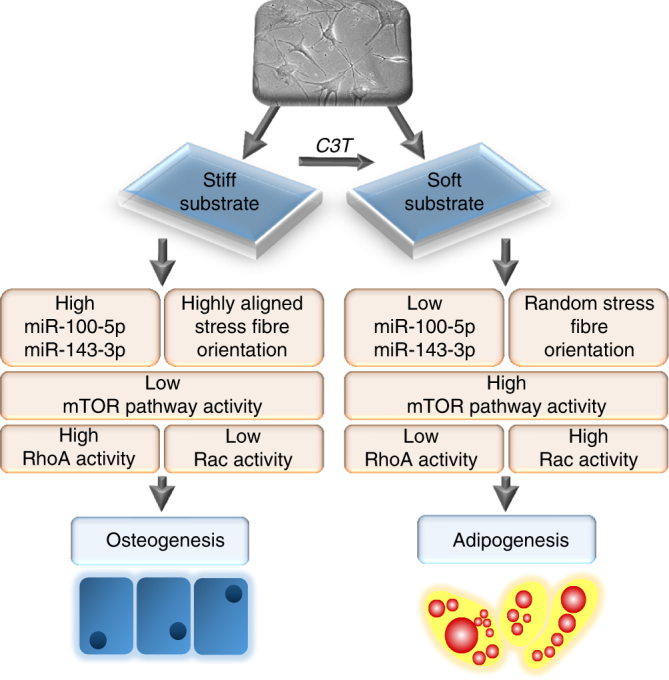
Proposed model of differential mTOR regulation and hMSC differentiation in response to miRNA signalling and substrate stiffness

Overall we determined that changes in miRNA expression correlate with differences in substrate stiffness and RhoA signalling, but were not observed to directly to regulate cytoskeletal architecture, instead acting upon downstream factors to impact upon MSC fate. This leads to a proposed mechanism for our findings whereby hMSCs on stiff substrates express high levels of *miR-100-5p* and *143-3p*, have decreased mTORC1 activity and increased osteogenic bias while hMSCs on soft substrates, or with RhoA inhibition, express low levels of *miR-100-5p* and *143-3p*, have increased mTORC1 activity and increased adipogenic bias (Fig. 7).

In accordance with this, we demonstrated greatly increased amounts of hydroxapatite accumulation when modulating *miR-100-5p* and *143-3p* in hMSCs encapsulated in 3D gelatin hydrogels. Importantly, this demonstrates that the modulation of the candidate miRNAs generated a mature osteoblast population and was not limited to initial commitment or effects on early osteogenic markers. Furthermore, this novel approach may have utility to promote hMSC differentiation when combined within biomaterial systems that have future clinical relevance. This is an important finding because effective control of hMSC fate within injectable hydrogels is necessary if they are to be used successfully in the clinic to regenerate new functional tissue.

Our data also provide new insights into cell mechanotransduction and the mechanisms that regulate the response of hMSCs to the surrounding extracellular environment. By providing the first systematic determination of changes to miRNA signalling in response to physical stimuli, we have demonstrated proof-of-concept that miRNA signalling links mechanotransduction to cell fate processes. Specifically, the targets of *miR-100-5p* and *143-3p* highlighted a new role for mTOR signalling in the mechano-regulation of hMSC fate. Given the growing body of research to indicate sensitivity of hMSCs to a wide variety of physical cues, there will undoubtedly be significant future findings regarding miRNA signalling networks that are sensitive to these other cues.

In summary, we have shown for the first time that miRNA signalling in hMSCs is modulated in response to significant variations in substrate stiffness, and that modulation of mechno-sensitive miRNAs provides an effective means to drive hMSC differentiation, even in the presence of mechanical cues expected to drive an alternate image. This provides new insights into the mechanisms that regulate hMSC fate, providing information of specific miRNAs as well as evidence of their mechanism of action via mTOR modulation. Furthermore, given the utility of small RNAs as inexpensive and relatively simple biomolecules to deliver, this study provides the first evidence of a novel avenue to drive hMSC fate for tissue regeneration and could have wide ranging applications in treating musculoskeletal disorders.

## Methods

### Substrate fabrication and functionalisation

Glass coverslips were treated with (3-Aminopropyl)triethoxysilane, fixed through a 30 min incubation in 1% glutaraldehyde, rinsed in dH_2_O and air dried. Glass slides were treated with chlorotrimethylsilane, rinsed under dH_2_O dried with nitrogen gas. PAM gels were crosslinked between the treated slides and coverslips, polymerised under nitrogen for 10 min, then left to swell in sterile PBS at 4 °C overnight. The composition of the PAM gels used is shown in Supplementary Table 10. Once swollen, gels were functionalised with 1 mg/mL sulfosuccinimidyl 5(4′-azido-2′-nitrophenyl-amino) hexanoate (sulfo-SANPAH, Abcam), extensively washed with PBS and incubated overnight in 25 µg/mL of Collagen-I (Gibco) diluted in PBS. PAM gels with Young’s modulus of 70 and 0.6 kPa are denoted as stiff and soft substrates, respectively.

### Rheological characteristics

The rheological properties of PAM gels were determined using Anton Paar Physica MCR 501 parallel plate Rheometer. PAM gels were placed in 8 mm diameter parallel plates separated by a 0.5 mm gap. Strain sweeps were conducted from 0.1–100% at a frequency of 50 rad/s. Frequency sweeps were performed at a constant strain of 1% and frequencies between 0.1 and 100 rad/s. Time sweeps were determined at 37 °C with a 1% strain and at an angular frequency of 6.28 rad/s.

### Mesenchymal stem cell culture

hMSCs (bone marrow-derived, Lonza) were cultured in DMEM-low glucose supplemented with 100 U/mL penicillin, 100 µg/mL streptomycin (DMEM/ps) and 10% fetal bovine serum (FBS) at 37 °C and 5% CO_2_. These MSCs are tested and certified to meet all criteria of and MSC as defined by the ISCT and are free from tested pathogens. All cultures were tested and confirmed free of mycoplasma every 3 months using a Mycoalert kit (Lonza). Prior to all experiments, cells were serum-starved overnight in DMEM/ps with 0.25% FBS. Cells were passaged at 80% confluency and seeded at a density of 2.5 × 10^3^ cells/cm^2^ for subsequent passaging and cells were used up to passage 6. Inhibitors were added to cell cultures at the following concentrations: C3T (RhoA inhibitor, 1 µg/mL) and Rapamycin (mTOR inhibitor, 50 nM).

### FRET biosensor analysis

One million hMSCs were transfected with 10 μg DNA using the 4D-Nucleofector™ System together with Human MSC Nucleofector® Kit (Lonza) on high-efficiency settings. Following nucleofection the cells were plated in DMEM/ps with 10% FBS overnight to recover before passaging onto PAM gels in DMEM/ps with 0.5% FBS. Images for analysis were collected on a LSM710 Zeiss confocal microscope equipped with a 40X water immersion objective (Zeiss, Jena, GER) using a heated stage at 37 °C with 5% CO_2_ atmosphere. CFP and FRET channels were recorded using a 458 nm laser line and collecting the emissions in the donor (BP 470e490 nm) and acceptor (BP 530e590 nm) emission regions, respectively. In addition, crosstalk and YFP channels were recorded using the 514 nm laser line for excitation and collecting the emission in the donor and acceptor emission regions. Images were acquired by sequential acquisition. For FRET measurements, a modified version of the FRET emission ratio was used to calculate this parameter on a pixel-by-pixel basis, as described previously^14^. The FRET index was calculated for every image as the average [FRET/Donor] emission ratio for selected regions of interest. Greater than 20 cells were tested per condition and repeated using multiple MSC donors.

### Osteo:adipogenic differentiation switch assay

hMSCs were plated onto the appropriate substrate at 5 × 10^3^ cells/cm^2^ and treated with mixed (1:1 adipogenic:osteogenic) inductive medium, with medium changes performed every 3 to 4 days. Adipogenic medium consisted of DMEM high-glucose with 10% FBS, 10 µg/mL insulin, 0.5 mM isobutyl-1-methylxanthine, 1 µg/mL dexamethasone and 0.2 mM indomethacin. Osteogenic medium consisted of DMEM low-glucose with 10% FBS, 50 µM ascorbate-2-phosphate, 100 ng/mL dexamethasone and 10 mM β-glycerophosphate.

At 14 days, differentiation was assessed via staining of ALP and Oil Red O (ORO) staining. To detect ALP activity, hMSCs were washed with PBS and incubated in 1 mg/mL Fast Blue and 0.2 mg/mL Napthol AS-MX Phosphate in 0.1 M Tris-HCl (pH 9.2) for 5 min at room temperature. Cells were then washed with PBS and fixed in 4% paraformaldehyde for 15 min. Subsequently, cells were stained using 0.5% ORO solution for 30 min. Images were obtained on a Nikon Eclipse Ti microscope equipped with Spot imaging software.

### miRNA sequencing and analysis

RNA was isolated using the Qiagen miRNeasy mini kit (Cat #217004). DNA was removed from Total RNA using a Qiagen RNase-Free DNase Set (Cat #79254), and RNA was run on an Agilent Bioanalyser and Thermo Nanodrop for QC purposes. An aliquot of 1 µg of Total RNA was used as input into a NEBNext Small RNA Library Prep Set for Illumina Kit (Cat #E7330), as per the manufacturer’s instructions. Libraries were loaded onto 2% agarose and electrophoresed at 100 V for 60 min and stained in 1× SYBR Gold for 15 min at RT with agitation. Bands corresponding to ~147 bp marker were excised, purified using a QIAGEN gel extraction kit, and re-run on a bioanalyser to confirm purity. Libraries were sequenced on an Illumina MiSeq and counted with qmiR against miRBase (v20). Differential expression determined using EdgeR with filtering criteria that miRNAs had to be expressed in two out of the three donors at a level of 10 counts per million or more to be included in the analysis. miRNAs were considered differentially expressed when the log_2_ fold change between conditions was greater than or equal to 2, and the FDR controlled p-value was less than 0.05. Targetscan was used to generate lists of target mRNAs for each of these differentially expressed miRNAs and these genes were analysed for functional enrichment using Ingenuity Pathway Analysis software.

### Quantitative real-time RT-PCR for miRNA

To isolate miRNA, total RNA was extracted using the Qiagen miRNeasy mini kit with on-column DNase treatment (Cat # 217004) according to the manufacturer’s instructions. cDNA was synthesised from 500 ng RNA using miScript II RT kit (Qiagen) in a total volume of 20 µl. Reverse transcription was performed in the Biorad T100 Thermal Cycler using the following cycling conditions: 60 min at 37 °C and 5 min at 95 °C. Quantitative PCR reactions for miRNA analysis were set-up in a total volume of 10 µL with miScript SYBR Green kit (Qiagen) and miScript primers (Qiagen). A CFX96 Real-Time System (Bio-Rad) was used to run the samples with cycling parameters of 15 min at 95 °C followed by 40 cycles of 15 s at 94 °C, 30 s at 55 °C and 30 s at 70 °C. The relative level of miRNA expression was calculated using the 2^–ΔΔCt^ method with *RNU6* as a reference.

### Quantitative real-time RT-PCR for mRNA

To analyse mRNA, total RNA was extracted using the Qiagen RNeasy mini kit with on-column DNase treatment (Cat #74104) according to the manufacturer’s instructions. cDNA was synthesised from up to 750 ng RNA using Superscript VILO (Thermo Scientific) in a total volume of 20 µl. Reverse transcription was performed in the Biorad T100 Thermal Cycler using the following cycling conditions: 10 min at 25 °C, 60 min at 42 °C and 5 min at 85 °C. Quantitative PCR reactions were set-up in a total volume of 10 µL with 1× ABI Fast SYBR Green Mastermix and 0.2 µM forward and reverse primers. The primer sequences were listed in Supplementary Table 11. A CFX96 Real-Time System (Bio-Rad) was used to run the samples with fast cycling parameters of 20 s at 95 °C, 3 s at 95 °C and 30 s at 60 °C, which was repeated for 40 cycles and followed by a melt curve. Data were analysed by the 2^–ΔΔCt^ method using *RPS27a* as a reference gene.

### miRNA modulation

To up/downregulate miRNA activity, hMSCs were treated with either human mercury LNA^TM^ miRNA mimics and mimic negative control or human antisense mercury LNA^TM^ miRNA inhibitors and miRNA inhibitor control respectively (Exiqon). Details of the mimics and inhibitors are listed in Supplementary Table 12. Cells were transfected using Lipofectamine RNAiMAx transfection reagent (Thermo Scientific) and 10 or 25 nM of miRNA mimic, miRNA inhibitor or scrambled miRNA control were added. Following transfection with miRNA mimics and inhibitors, hMSCs were plated onto PAM gels for RNA/protein isolation or plated into soft and stiff PAM gels at a density of 5 × 10^3^ cells/cm^2^ for differentiation analysis. For rapamycin experiments, MSC were treated with 50 nM rapamycin (Cayman Chemical) for the duration of the culture period.

### mTOR (FRAP1) reporter assay

A FRAP1 (mTOR) reporter construct (HmiT006426 3′UTR, Genecopoeia™) was transiently transfected into 1 × 10^6^ hMSCs (2 µg) using a 4D Nucleofector with associated Human MSC Nucleofector Kit (Lonza). Following transfection, cells were incubated under standard culture conditions for 12 hrs, re-distributed across a 96-well plate at a density of 4 × 10^3^ cells/cm^2^ and incubated a further 12 h under standard culture conditions.

Selected miRNA and mimics and inhibitors of *miR-100-5p* were added to transfected hMSCs using the lipofectamine RNAiMAX system (Invitrogen) as above and incubated for 24 h and reporter gene activity measured using the Secrete-Pair™ Dual Luminescence Assay Kit (GeneCopoeia) on a CLARIOstar® microplate reader (BMG labtech). Results were calculated as the ratio of luciferase light units to secreted ALP.

### Western blotting

Cell lysates were prepared in RIPA buffer, supplemented with 1% protease inhibitor cocktail (Roche). Lysates were subsequently combined with 5× Laemmli sample buffer and assessed through SDS-PAM gel electrophoresis (SDS-PAGE) and subsequent western blot analysis^52,53^. To ascertain complete separation for proteins of interest, pre-cast 4–12% gradient PAM gels (Thermo Scientific) were applied. Following electrophoresis, proteins were transferred to PVDF membrane under wet electro-transfer conditions using transfer buffer ([10%] ethanol, 25 mM Tris, 0.1% SDS and 190 mM glycine in H_2_O) maintained at 350 mA for 1 h (at 4 °C). Membranes were blocked in PBS and 5% skim milk powder at RT for 30 min and incubated overnight in primary antibody (diluted in blocking solution) at 4 °C; mTOR [1:1000] (Abcam ab87540), β-Tubulin [1:1000] (Sigma T8328), GAPDH [1:2000] (Millipore MAB374), LARP1 [1:3000] (Abcam ab86539), RICTOR [1:1000] (Abcam ab104838), RAPTOR [1:2000] (Abcam ab5454), Akt [1:1000] (Cell Signalling 4685S), Phosphorylated Akt [1:1000] (Cell Signalling 4058S). Membranes were incubated in secondary antibody for 1 h at RT; goat anti-rabbit conjugated POD [1:3000] (Abcam ab6721), goat anti-mouse conjugated POD [1:4000] (Abcam ab6728). Chemiluminescence signal was detected using Pierce ECL Plus solution (Thermo Fisher) and a UVITEC mini HD6 detector. Original western blot images are shown in Supplementary Fig. 8.

### 3D gelatin-PEG gels

hMSCs were transfected with miRNA mimics/inhibitors for 24 h prior to encapsulation in gelatin-PEG hydrogels^24^. Cells were harvested by TrypLE^™^ Express treatment, spun down and resuspended at a density of 1.25 × 10^4^ cells/μL. Hydrogel stock solutions of 10% (w/v) GelNB (gelatin norbornene), 15% (w/v) PEG(SH)_2_ (poly(ethylene glycol) dithiol (Mn 2000 Da))and 5% (w/v) LAP (lithium phenyl-2,4,6-trimethylbenzoylphosphinate) photoinitiator were freshly prepared in sterile DPBS. GelNB stock solutions were incubated at 37 °C to ensure the solubility and homogenisation. Gel stock solutions (4% (w/v) GelNB, 1% (w/v) PEG(SH)_2_, 0.03% (w/v) LAP) and corresponding cell suspensions were combined to deliver the final 5% (w/v) hydrogel with hMSCs at 2.5 × 10^3^ cells/μL. Triplicate drops (25 μL/drop) per condition were pipetted into a petri dish (Cellvis) and exposed to the visible light (400~500 nm, 10 mW/cm^2^) for 10 min. The cured hMSC-laden samples were rinsed thrice with 1.5 mL media and cultured in the osteogenic media for 14 days with media change every 3 days. To stain hydroxyapatite, gels were washed with PBS, fixed with 4% paraformaldehyde and incubated with Osteoimage (Lonza) as per the manufacturer’s instructions, followed by incubation in Hoechst to label cell nuclei. Fluorescence detection and 3D projections were obtained using a Zeiss LSM 780 confocal microscope and processed using Zen software. Image analysis was performed on multiple images per gel, normalising the intensity of the Osteoimage staining to Hoechst to account for any variability on cell number.

### Statistical analysis

A Kolmogorov–Smirnov test was used to test data for Normal distribution and Levene’s test used to determine homogeneity of variance. Data with a Normal distribution were analysed by one-way ANOVA and Tukey (equal variance) or Games-Howell (unequal variance) post hoc tests. Non-parametric data were analysed by Kruskal–Wallis test. All statistical analysis was performed using SPSS v23.

### Data availability

The authors declare that all data supporting the findings of this study are available within the article and its Supplementary information files or from the corresponding author upon reasonable request.

## Electronic supplementary material

Supplementary Information

Description of Additional Supplementary Files

Supplementary Data 1

Supplementary Data 2

Supplementary Data 3
