# Supplementary material for: Mechanically-sensitive miRNAs bias human mesenchymal stem cell fate via mTOR signalling
Source: Nat Commun. 2018 Jan 17;9:257. doi: 10.1038/s41467-017-02486-0 (PMC5772625; doi:10.1038/s41467-017-02486-0)
Supplement: Supplementary file 1 — Supplementary Information [file 41467_2017_2486_MOESM1_ESM.pdf]

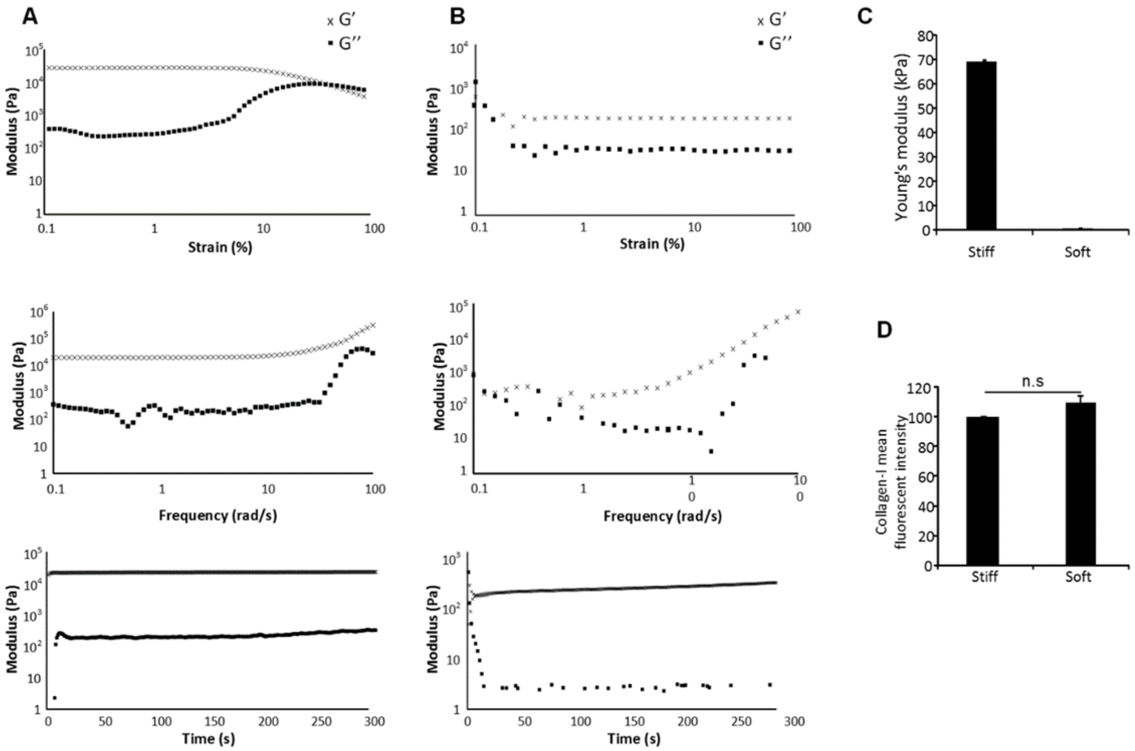

Supplementary Figure 1. Rheological characterization of polyacrylamide (PAM) gels. Strain, frequency and time sweeps of **A)** stiff and **B)** soft PAM gels showing storage (G’) and loss (G’’) moduli. **C)** Young’s modulus of soft and stiff PAM gels. Data is shown as mean ± SEM for n=12. **D)** Mean fluorescent intensity of collagen-I immunolocalisation. Data is shown as mean ± SEM for n>15 images taken across 3 separate gels.

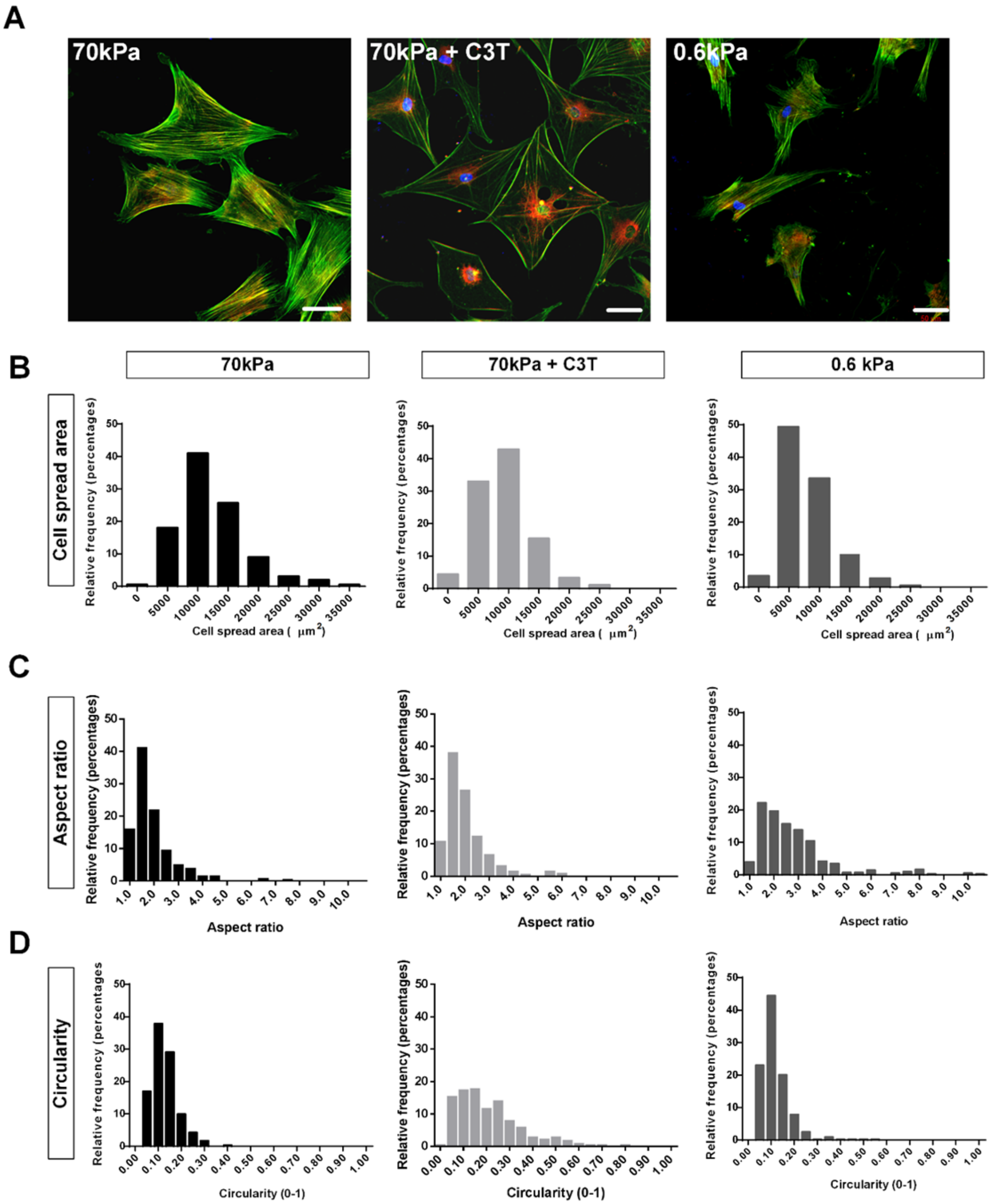

Supplementary Figure 2. Analysis of morphology in response to substrate stiffness and RhoA signalling. A) Staining of hMSC structure showing actin (green), nuclei (blue) and non-muscle myosin II (red). Scale bar, 50 μm. Histograms showing **B)** cell spread area, **C)** aspect ratio and **D)** circularity for populations of >50 MSCs per condition.

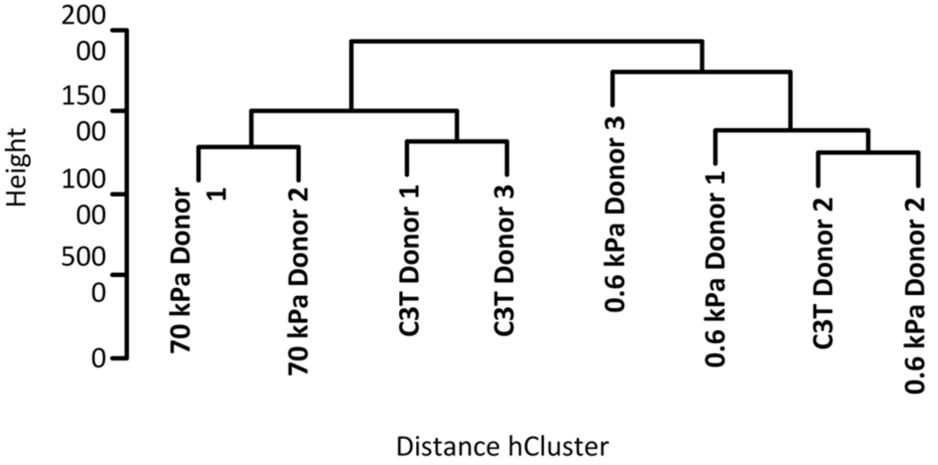

Supplementary Figure 3. Hierarchical clustering of miRNA sequencing samples.

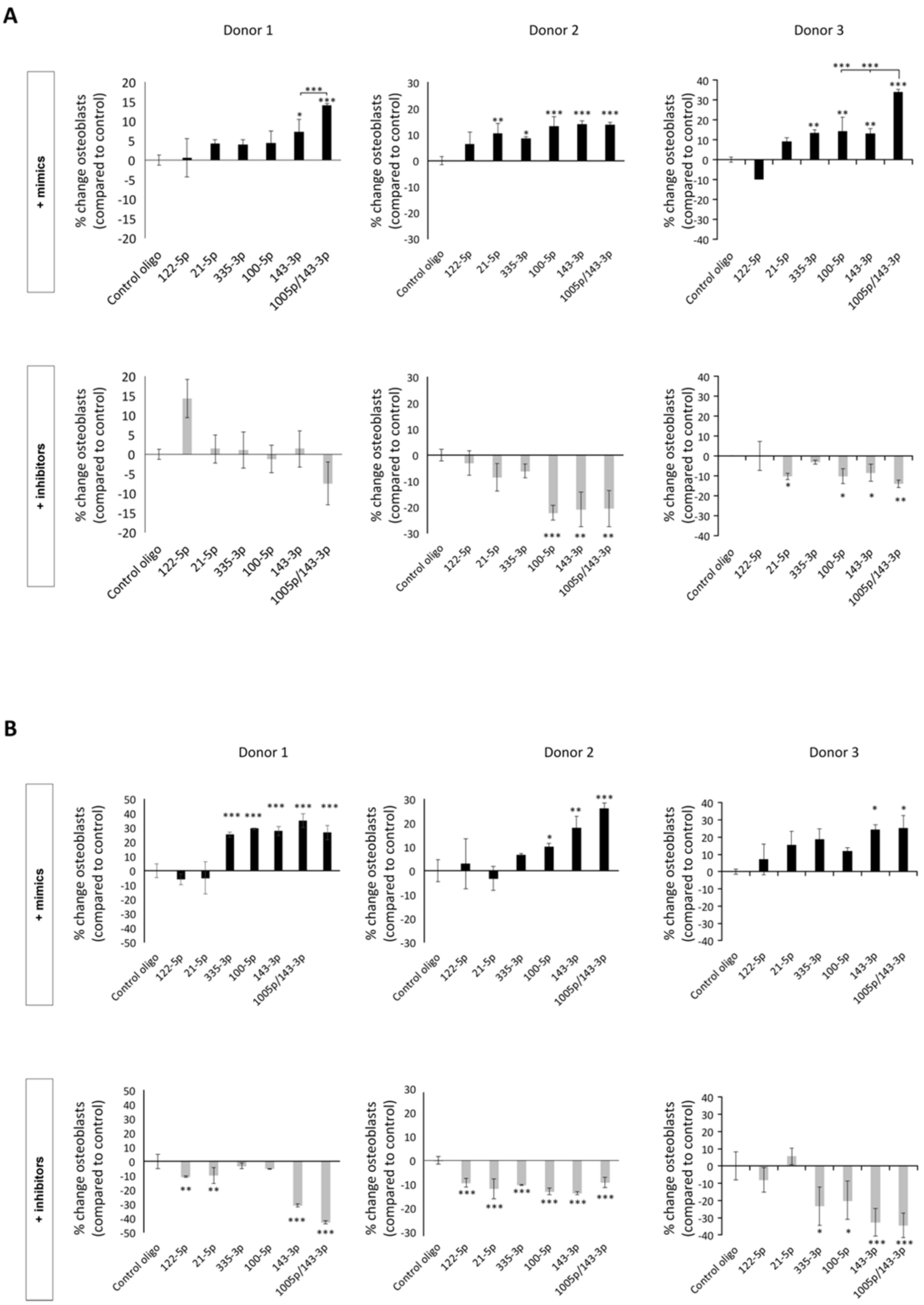

Supplementary Figure 4. Effect of miRNA modulation on MSC differentiation bias for individual MSC donors. Effect of miRNA mimics and inhibitors on MSCs cultured on **A)** 70kPa and **B)** 0.6kPa substrates. Data is shown as mean change in proportion of osteoblasts relative to control ± SD. Statistically different samples are denoted by $p < 0.05$ (*), 0.01 (**) and 0.001 (***).

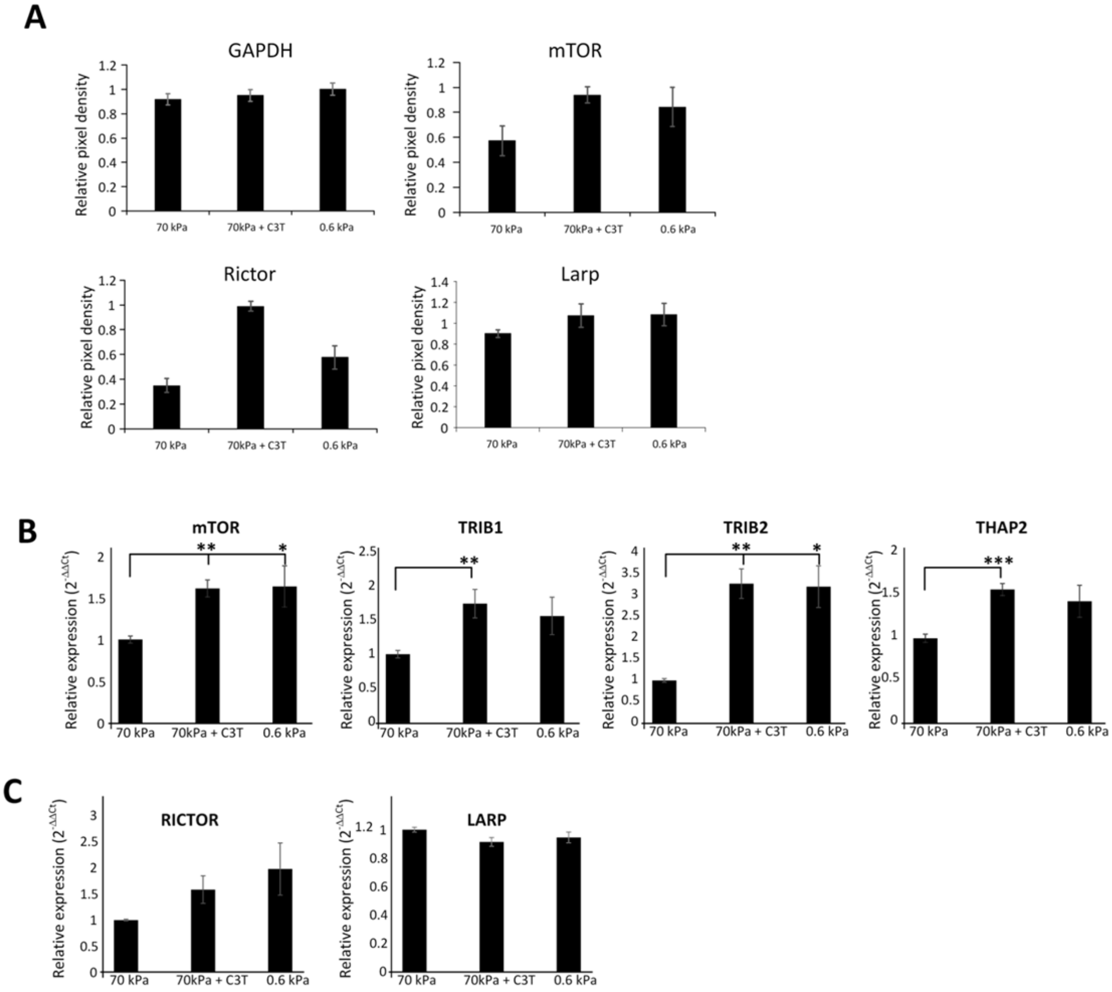

Supplementary Figure 5. Expression analysis of mTOR pathway components in response to substrate stiffness and RhoA signalling. A) Quantification of proteins in MSCs cultured on 70 kPa, 70 kPa + C3T or 0.6 kPa substrates. Data are expressed as relative pixel density units, all graphs show mean ± SEM for 3 independent hMSC donors. qPCR determination of the relative expression of miRNA target genes for B) *miR-100-5p* and C) *miR-143-3p*. All data is represented as mean ± SEM relative to expression in 70 kPa samples. Statistically different samples are denoted by $p < 0.05$ (*), 0.01 (**) and 0.001 (***).

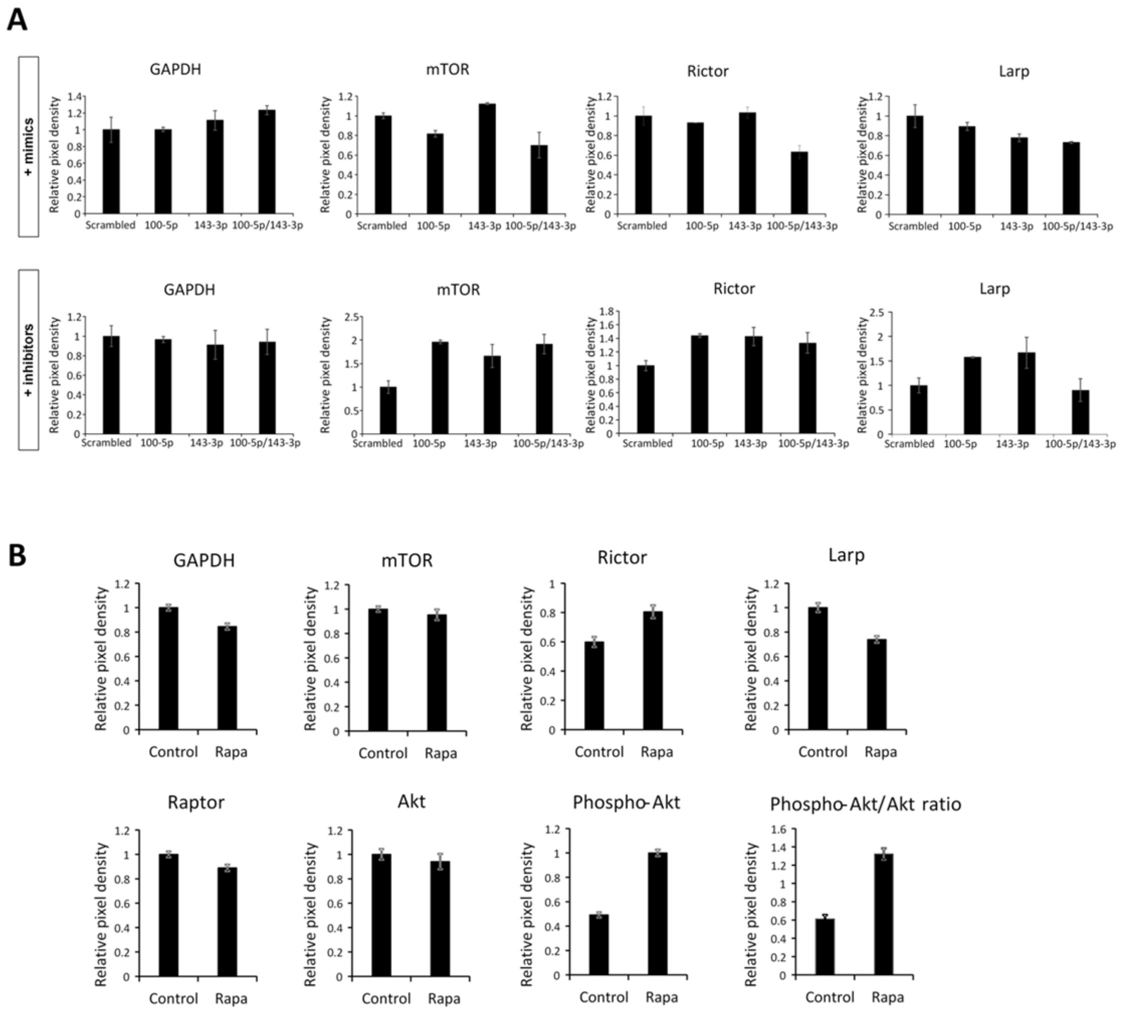

Supplementary Figure 6. Expression analysis of mTOR pathway components in response to miRNA mimics/inhibitors or rapamycin treatment. Quantification of proteins in **A)** MSCs transfected with miRNA mimics or inhibitors and **B)** MSCs treated with 50 nM rapamycin. Data are expressed as relative pixel density units, all graphs show mean ± SEM for 3 independent hMSC donors.

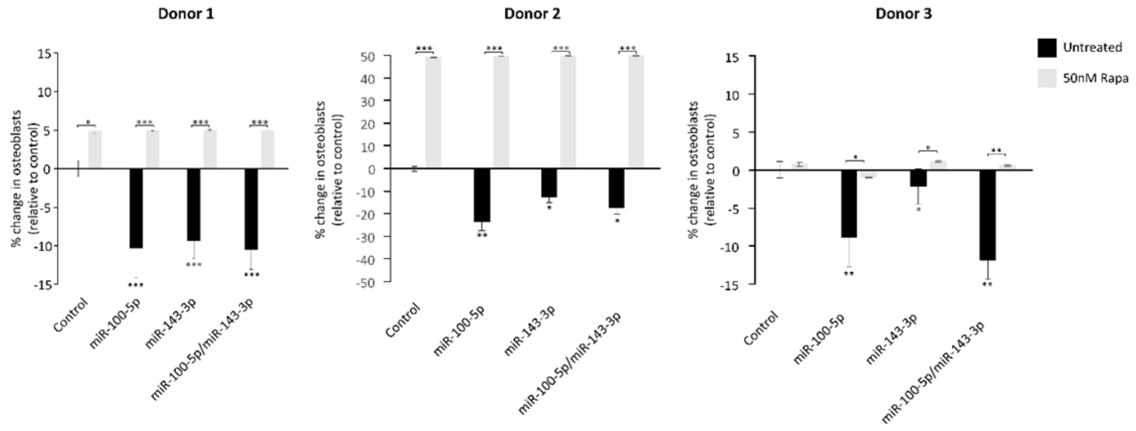

Supplementary Figure 7. Opposing effects of miRNA inhibitors and Rapamycin on MSC differentiation bias. Data shown for 3 independent MSC donors for untreated (black bars) and rapamycin treated (grey bars) MSCs. Data is shown as mean change in proportion of osteoblasts relative to control ± SD. Statistically different samples are denoted by $p < 0.05$ (*), 0.01 (**) and 0.001 (***).

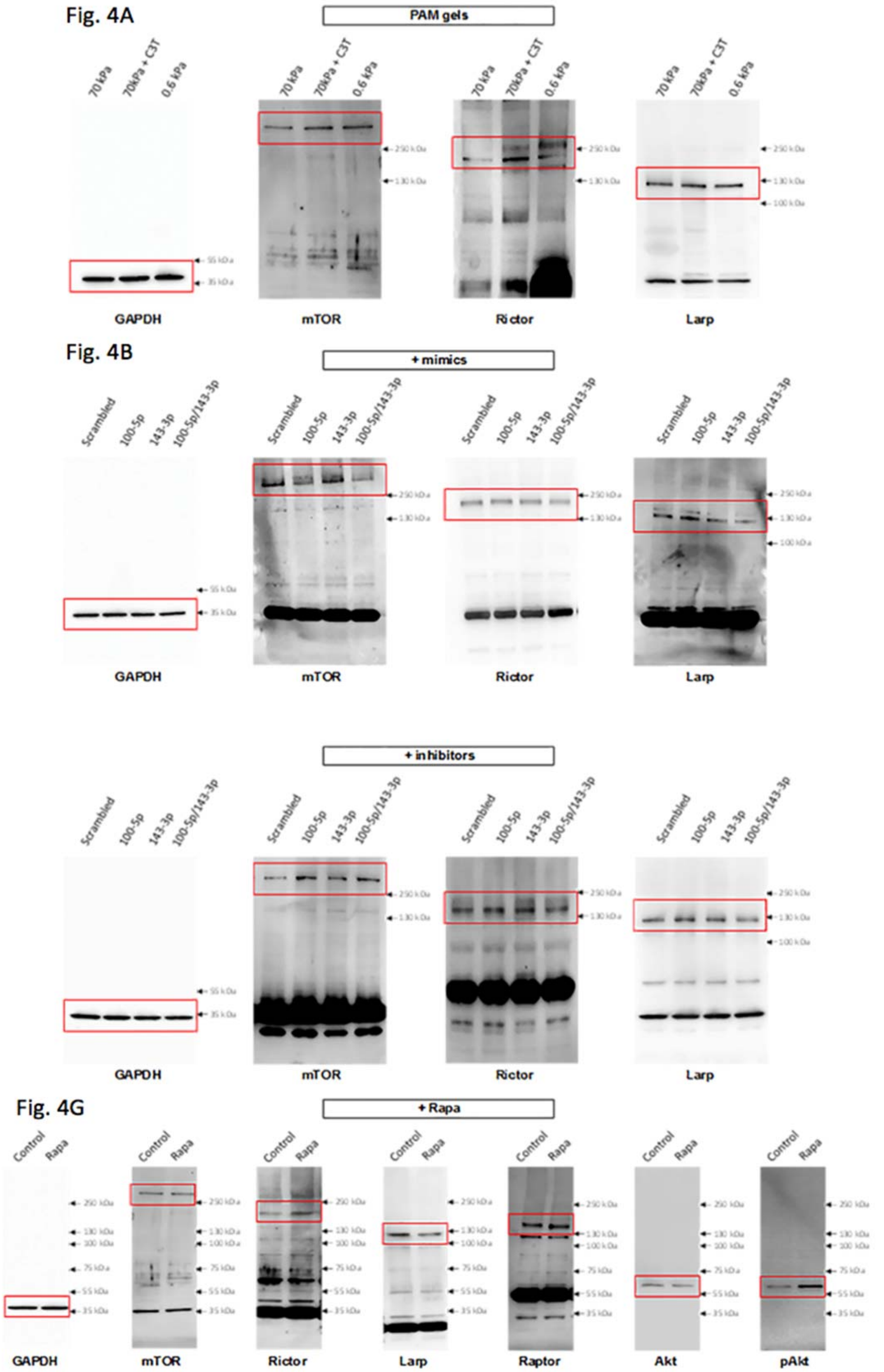

Supplementary Figure 8. Full scans of uncropped Western blots presented in the main figure.

Supplementary Table 1. miRNAs upregulated in 70 kPa + C3T vs 70 kPa.

miRNA	logFC	logCPM	P Value	FDR
hsa-mir-3929-3p	5.5759784	5.353010	1.101922e-13	5.774073e-11
hsa-mir-3929	4.4738797	5.569399	8.551213e-12	2.240418e-09
hsa-mir-5096-3p	2.5543970	5.970366	3.714409e-07	4.865875e-05
hsa-mir-619-5p	4.1287945	7.014513	3.289117e-06	3.173089e-04
hsa-mir-566-5p	2.7488159	4.667838	7.156853e-06	5.357416e-04
hsa-mir-1285-1-5p	3.4135330	5.047673	1.481225e-04	9.702022e-03
hsa-mir-5096	2.8533990	4.208759	2.562158e-04	1.464287e-02
hsa-mir-7851-5p	5.5239765	5.879177	2.794442e-04	1.464287e-02
hsa-mir-5096-5p	2.0439955	5.276739	3.451199e-04	1.644026e-02
hsa-mir-1273d-3p	2.3882644	4.214999	4.330409e-04	1.890945e-02
hsa-mir-3159-3p	2.0467889	5.990651	6.318541e-04	2.364939e-02
hsa-mir-4419a	1.9017001	4.599033	7.185424e-04	2.510108e-02
hsa-mir-483-5p	2.2338434	4.222222	1.530696e-03	4.595441e-02
hsa-mir-566-3p	2.3271466	5.025884	1.578587e-03	4.595441e-02

Supplementary Table 2. miRNAs downregulated in 70 kPa + C3T vs 70 kPa.

miRNA	logFC	logCPM	P Value	FDR
hsa-mir-335-3p	-1.7669778	8.529429	3.214541e-08	5.614732e-06
hsa-mir-100-5p	-0.2792123	17.803562	3.633308e-06	3.173089e-04
hsa-mir-7974-3p	-2.6368929	4.672177	5.227040e-04	2.106899e-02
hsa-mir-1268b-5p	-1.6934567	6.626036	9.941078e-04	3.255703e-02

Supplementary Table 3. miRNAs upregulated in 0.6 kPa vs 70 kPa.

miRNA	logFC	logCPM	P Value	FDR
hsa-mir-122-5p	3.245741	5.981104	1.452461e-08	7.610898e-06
hsa-mir-4448-3p	1.444811	9.031696	1.041214e-04	2.727979e-02

Supplementary Table 4. miRNAs upregulated in 0.6 kPa vs 70 kPa + C3T.

miRNA	logFC	logCPM	P Value	FDR
hsa-mir-100-5p	0.2974540	17.803562	8.222047e-08	8.616706e-06
hsa-mir-6131-3p	1.0783050	15.120294	3.546137e-06	2.322720e-04
hsa-mir-4448-3p	1.5794037	9.031696	2.989968e-05	1.424312e-03
hsa-mir-1180-3p	0.9887903	8.566305	7.971763e-05	3.213234e-03
hsa-mir-7706-3p	1.6518474	6.740006	2.252161e-04	6.211222e-03
hsa-mir-25-5p	2.0540046	5.784007	5.785608e-04	1.263191e-02
hsa-mir-3615-3p	0.8177897	8.702686	6.258842e-04	1.311853e-02
hsa-mir-122-5p	1.6418679	5.981104	1.083907e-03	1.893225e-02
hsa-mir-3687-3p	1.9865586	5.323219	1.959968e-03	3.209447e-02
hsa-mir-320b-2-3p	0.7504807	8.693615	2.212632e-03	3.410056e-02
hsa-mir-7974-3p	2.3248083	4.672177	3.574127e-03	4.928533e-02

Supplementary Table 5. miRNAs downregulated in 0.6 kPa vs 70 kPa + C3T

miRNA	logFC	logCPM	P Value	FDR
hsa-mir-3929-3p	-4.4900459	5.353010	1.024551e-12	3.465906e-10
hsa-mir-3929	-4.2621572	5.569399	1.322865e-12	3.465906e-10
hsa-mir-619-5p	-4.2848277	7.014513	2.453039e-08	4.284641e-06
hsa-mir-5096-3p	-2.5041362	5.970366	4.044641e-08	5.298480e-06
hsa-mir-566-5p	-3.2134090	4.667838	1.694976e-07	1.480279e-05
hsa-mir-5096	-3.8722842	4.208759	8.528787e-07	6.384406e-05
hsa-mir-21-5p	-0.6454914	16.389694	8.286239e-06	4.824433e-04
hsa-mir-5096-5p	-2.3107125	5.276739	1.906115e-05	9.988041e-04
hsa-mir-3159-3p	-2.1983740	5.990651	4.601951e-05	2.009519e-03
hsa-mir-660-5p	-2.0389909	6.124728	9.497978e-05	3.554958e-03
hsa-mir-1273d-3p	-2.5101363	4.214999	1.328769e-04	4.641833e-03
hsa-mir-1285-1-5p	-2.9187077	5.047673	1.518824e-04	4.974150e-03
hsa-mir-7851-5p	-4.5803584	5.879177	1.868686e-04	5.618112e-03
hsa-mir-382-3p	-1.4297022	7.143209	1.929886e-04	5.618112e-03
hsa-mir-136-3p	-2.5662955	4.665143	3.089401e-04	7.978467e-03
hsa-mir-337-3p	-1.5090304	6.466167	3.197477e-04	7.978467e-03
hsa-mir-146a-5p	-2.2848487	6.080851	4.821789e-04	1.148462e-02
hsa-mir-186-5p	-1.0251221	7.834047	5.105812e-04	1.163237e-02
hsa-mir-140-5p	-1.1710280	-1.1710280	7.787207e-04	1.569422e-02
hsa-mir-1273a-5p	-2.0655408	4.491878	8.283748e-04	1.607661e-02
hsa-mir-21-3p	-1.4118082	6.715625	8.713034e-04	1.630582e-02
hsa-mir-376c-3p	-1.7264774	5.206281	9.740389e-04	1.759988e-02
hsa-mir-34a-5p	-1.2188259	7.278523	1.678721e-03	2.837581e-02

hsa-mir-494-3p	-0.8668309	8.376215	2.132062e-03	3.385456e-02
hsa-mir-195-5p	-1.7854635	4.869119	2.437922e-03	3.649918e-02
hsa-mir-8086-3p	-2.5630816	6.122087	3.222029e-03	4.689842e-02
hsa-mir-656-3p	-2.1238426	4.190869	3.449753e-03	4.885597e-02

Supplementary Table 6. miRNAs upregulated in [70 kPa + C3T and 0.6 kPa] vs 70 kPa.

miRNA	logFC	logCPM	P Value	FDR
hsa-mir-3929-3p	4.34775	5.35301	3.03E-09	1.59E-06
hsa-mir-3929	3.2717	5.569399	9.51E-08	2.49E-05
hsa-mir-122-5p	2.770195	5.981104	7.97E-07	*1.04E-04*
hsa-mir-619-5p	2.915437	7.014513	2.79E-04	2.92E-02
hsa-mir-5096-3p	1.583912	5.970366	5.19E-04	4.53E-02

Supplementary Table 7. miRNAs downregulated in [70 kPa + C3T and 0.6 kPa] vs 70 kPa.

miRNA	logFC	logCPM	P Value	FDR
hsa-mir-335-3p	-1.236386	8.529429	3.26E-07	5.69E-05

Supplementary Table 8. miRNAs upregulated in 0.6 kPa vs [70 kPa and 70 kPa + C3T].

miRNA	logFC	logCPM	P Value	FDR
hsa-mir-3929-3p	3.433864	5.35301	5.61E-09	1.97E-06
hsa-mir-3929	3.2540579	5.569399	7.50E-09	1.97E-06
hsa-mir-619-5p	3.3575916	7.014513	1.81E-06	1.58E-04
hsa-mir-21-5p	0.5578685	16.389694	7.11E-06	5.32E-04
hsa-mir-5096-3p	1.6641658	5.970366	4.96E-05	3.25E-03
hsa-mir-566-5p	2.2932767	4.667838	8.58E-05	5.00E-03
hsa-mir-660-5p	1.7911026	6.124728	1.23E-04	6.42E-03
hsa-mir-5096	2.960036	4.208759	1.35E-04	6.42E-03
hsa-mir-382-3p	1.171821	7.143209	4.40E-04	1.92E-02
hsa-mir-136-3p	2.1956999	4.665143	4.82E-04	1.94E-02
hsa-mir-494-3p	0.7947635	8.376215	1.08E-03	3.76E-02
hsa-mir-5096-5p	1.5545034	5.276739	1.29E-03	4.13E-02
hsa-mir-7851-5p	3.6124295	5.879177	1.34E-03	4.13E-02
hsa-mir-21-3p	1.1892173	6.715625	1.43E-03	4.17E-02
hsa-mir-143-3p	0.6391022	15.017312	1.70E-03	4.46E-02
hsa-mir-337-3p	1.144301	6.466167	2.06E-03	4.75E-02
hsa-mir-186-5p	0.7785284	7.834047	2.08E-03	4.75E-02

Supplementary Table 9. miRNAs downregulated in 0.6 kPa vs [70 kPa and 70 kPa + C3T]

miRNA	logFC	logCPM	P Value	FDR
hsa-mir-122-5p	-2.3200241	5.981104	5.40E-08	9.44E-06
hsa-mir-4448-3p	-1.5091103	9.031696	3.32E-07	4.34E-05
hsa-mir-6131-3p	-0.9025831	15.120294	1.21E-06	1.26E-04
hsa-mir-100-5p	-0.1509109	17.803562	9.99E-04	3.74E-02
hsa-mir-320b-2-3p	-0.6169668	8.693615	1.60E-03	4.41E-02
hsa-mir-320a-5p	-1.3021374	6.746814	2.05E-03	4.75E-02

Supplementary Table 10. Composition for polyacrylamide (PAM) hydrogels.

	**Soft (0.6 kPa) PAM gels**	**Stiff (70 kPa) PAM gels**
Acrylamide [30%]	50 µl	200 µl
Bis-Acrylamide [1%]	75 µl	300 µl
dH_2O	375 µl	N/A
TEMED	1.25 µl	1.25 µl
APS [10%]	3.75 µl	3.75 µl

APS: ammonium persulfate. TEMED: N, N, N’, N’-Tetramethylethylenediamine.

Supplementary Table 11. qPCR forward and reverse primer sequences used in this study.

	**Forward 5’-3’**	**Reverse 3’-5’**
GAPDH	ATGGGGAAGGTGAAGGTCG	TAAAAGCAGCCCTGGTGACC
RPS27a	TGGATGAGAATGGCAAAATTAGTC	CACCCCAGCACCACATTCA
mTOR	CAGAAGGTGGAGGTGTTTGAG	TGACATGACCGCTAAAGAACG
TRIB1	TTGGGGACATGCACTCCTATG	GGCGGAGACAATCTGCTTGA
TRIB2	GACTCCGAACTTGTCGCATTG	GGCACGAAAAACGTGGTCT
THAP2	GGCTGTGCCACTACCTACAAC	CAGGCGAACCCATTCTTTTCTT
RICTOR	AGAAGCACGATTTCTAGCCAGT	AGTAGACCTCGCCTTATTTCCA
LARP1	GGTTCCAGCAAGTTCCTAC	ATCCAGTGTCCGTCCTTC

GAPDH: glyceraldehyde-3-phosphate dehydrogenase. RPS27a: ribosomal protein S27a. mTOR: mechanistic target of rapamycin. TRIB1: tribbles pseudokinase 1. TRIB2: tribbles pseudokinase 2. THAP2: THAP domain containing 2. RICTOR: RPTOR independent companion of MTOR complex 2. LARP1: La ribonucleoprotein domain family member 1.

Supplementary Table 12. Details of Exiqon miRNA mimics and miR inhibitors used in this study.

	**miRNA mimic**	**miRNA inhibitor**
Mimic negative control, FAM labelled	479995-011	-
miRNA inhibitor control, FITC labeled	-	199006-011
hsa-miR-100-5p	472477-001	4104079-001
hsa-miR-122-5p	470430-001	4104657-001
hsa-miR-143-3p	470035-001	4101245-001
hsa-miR-21-5p	473093-001	4102261-001
hsa-miR-335-3p	472642-001	4103230-001
