# Supplementary material for: Mechanically-sensitive miRNAs bias human mesenchymal stem cell fate via mTOR signalling
Source: Nat Commun. 2018 Jan 17;9:257. doi: 10.1038/s41467-017-02486-0 (PMC5772625; doi:10.1038/s41467-017-02486-0)
Supplement: Supplementary file 2 — Description of Additional Supplementary Files [file 41467_2017_2486_MOESM2_ESM.pdf]

Description of Additional Supplementary Files

File Name: Supplementary Data 1
Description: “Canonical pathways” enriched in predicted targets of differentially expressed miRNAs between 70 kPa and 70 kPA + C3T.

File Name: Supplementary Data 2
Description: “Canonical pathways” enriched in predicted targets of differentially expressed miRNAs between 70 kPa and 0.6 kPa.

File Name: Supplementary Data 3
Description: “Canonical pathways” enriched in predicted targets of differentially expressed miRNAs between 0.2kPa and 40kPa + C3T.
